# Comparison of Methods for the Estimation of the Maximum Oxygen Uptake of Men Drug Addicts

**DOI:** 10.3389/fphys.2021.683942

**Published:** 2021-09-10

**Authors:** Kun Wang, Haonan Jiang, Tingran Zhang, Lian Yin, Xi Chen, Jiong Luo

**Affiliations:** ^1^Research Centre for Exercise Detoxification, College of Physical Education, Southwest University, Chongqing, China; ^2^Chongqing Drug Rehabilitation Administration, Chongqing, China

**Keywords:** men, drug addicts, maximum oxygen uptake, cardiopulmonary exercise test, indirect measurement method

## Abstract

**Background:** Maximum oxygen uptake (VO_2max_) is an important respiratory physiological index of the aerobic endurance of the body, especially for special groups such as drug addicts, and it is an important indicator for assessing the cardiopulmonary function and formulating exercise prescriptions. Although the cardiopulmonary exercise test (CPX) is a classic method to directly measure VO_2max_, this method is limited by factors such as cumbersome operating procedures and expensive equipment, resulting in its relatively low applicability. Recently, many studies have begun to focus on the estimation of VO_2max_ in different groups of people, but few studies have focused on drug addicts.

**Methods:** Fifteen chemically synthesized drug addicts (such as amphetamines) and Fifteen plant-derived drug addicts (such as heroin) were recruited at the Chongqing Compulsory Isolation and Drug Rehabilitation Center in China. First, the VO_2max_ of subjects was directly measured through the CPX. Second, after subjects were fully rested, they were required to complete the 30-s high-leg raise, 1,000-m walk, and 3-min step experiment. Finally, SPSS 21.0 software was used to perform the correlation and linear regression analysis to verify the estimated effectiveness.

**Results:** (1) Regardless of chemically synthesized or natural plant-derived drug addicts, the years of drug use and walking time of 1,000 m were significantly negatively correlated with VO_2max_ (chemically synthesized: *P* < 0.01 and natural plant-derived: *P* < 0.05), the number of 30-s high-leg raises was a significantly positive correlation with VO_2max_ (*P* < 0.05 and *P* < 0.01), and the 3-min step index was significantly positively correlated with VO_2max_ (*P* < 0.01 and *P* < 0.01). (2) Regression analysis shows that the 30-s high-leg lift, 1,000-m walking, and 3-min step experiment could effectively estimate the VO_2max_ of chemically synthesized and natural plant-derived drug addicts. (3) Multiple linear regression constructed by the years of drug use combined with the step index has the highest estimated accuracy for the VO_2max_ of chemically synthesized drug addicts (96.48%), while the unary regression equation established by a single step index has the highest prediction accuracy for the VO_2max_ of natural plant-derived addicts (94.30%).

**Conclusion:** The indirect measurement method could effectively estimate the VO_2max_ of drug addicts, but different measurement methods have certain differences in the estimation accuracy of VO_2max_ of different drug addicts. In the future, the physical characteristics of drug users can be fully considered, combined with more cutting-edge science and technology, to make the estimation accuracy of VO_2max_ closer to the real level.

## Introduction

Drug abuse (i.e., illegal substances) is a global problem that threatens public health and undermines social productivity. In China, at the end of 2018, there were 2.404 million drug users, accounting for 0.18% of the total population of the country (National Anti-Drug Committee Office, 2019). Although the growth rate has reduced, the total population of drug abusers was still huge. Drug abuse will cause serious damage to the psychological and physiological health of drug users. For example, the long-term drug use behavior was considered not only to cause loss of appetite, weakness of spirit, and dysfunction in brain regions such as forehead leaves and almond nucleus, but also led to different degrees of influence of respiratory, cardiovascular, and immune systems (Li, 2001; Huang et al., 2010; Ozburn et al., 2015; Zarrabi et al., 2016). In addition, according to the composition and harm of drugs, amphetamine-based chemically compounds are usually called chemically synthesized drugs, while heroin-based plant extracts are called natural plant-derived drugs (Xue, 2010; Feng et al., 2019). There are certain differences in the damage of different types of drugs to individuals. The chemically synthesized drugs mainly damage the central nervous system, and they easily cause mental damage and cognitive impairment (Panenka et al., 2013), while natural plant-derived drugs mainly damage the immune function and easily damage the heart and lungs or other internal organs (Hu, 1992; Ye, 1995). However, previous studies tend to focus on the mental (Stogner et al., 2014; Wang et al., 2019) and brain health (van Holst and Schilt, 2011; Chen S. J. et al., 2017) of drug abusers, and there were relatively few studies on the cardiopulmonary fitness of different drug addicts.

Maximum oxygen uptake (VO_2max_) refers to the amount of oxygen that the human body could take in per kilogram of body weight in 1 min during maximum or exhausting exercise. It was an important indicator for assessing individual aerobic endurance and cardiopulmonary function (Katzmarzyk et al., 2004) and also a crucial indicator for formulating aerobic exercise prescriptions for special groups such as drug addicts. After the American Heart Association listed cardiorespiratory endurance as the “fifth most important” vital sign in 2016, the importance of VO_2max_ has received further attention (Coquart et al., 2016). The classic method of evaluating and measuring VO_2max_ was to directly collect the breathing gas of the subject during exhausting exercise through a cardiopulmonary exercise test (CPX) and then calculate the VO_2max_ by the cardiopulmonary function test system. The data measured by CPX have high credibility and were considered as the “gold standard” for measuring VO_2max_, but it also has disadvantages such as expensive instruments, difficulty to carry, cumbersome operating procedures, and high requirements for the test environment, and there were potential safety risks for special people such as the old, the weak, the sick, and the young (Liu and Dong, 2013; Dong et al., 2017). For the compulsory drug addicts, it was quite difficult to directly measure the VO_2max_ of them due to their particularities, environment, equipment, and other internal and external factors. However, the effective assessment and measurement of VO_2max_ levels of drug addicts were crucial for formulating personalized and effective exercise prescriptions and improving the cardiopulmonary function of drug addicts.

The indirect measurement method refers to the conversion of the measured value into many directly measurable quantities, and the measurement results were calculated by the mathematical formulae. At present, there were many indirect measurement methods for VO_2max_, and the common indirect measurement methods include 20-m turn back run (Han, 2010; Paradisis et al., 2014), Cooper's 12-min run test (CRT) (Cooper, 1968), 6-min walk test (Ross et al., 2010), 6-min stair climbing (Guo et al., 2016), 12-min walk test (Kittredge et al., 1994), 1,000-m run (Li et al., 2010), and step test (Guo et al., 2005). Some studies use CRT to estimate the cardiopulmonary function of special groups such as asthma and obese children (Calders et al., 2008; Weisgerber et al., 2009). Meanwhile, studies have suggested that some indirect measurement methods have certain disadvantages. For example, the 20-m reentry run and CRT have a large load, the exercise load of the latter was more difficult to control (Dong et al., 2017), and the 6-min upstairs and downstairs have a moderate load but were prone to falls. In contrast, the step test has become a common indirect measurement method of VO_2max_ due to its low requirements for equipment and test sites (Dong et al., 2017; Fan et al., 2019). Meanwhile, the 30-s high-leg raise test was similar to the step test, which was suitable for large-sample group testing and was convenient for promotion. The load of 1,000-m walking was moderate, the operation method was simple, and the safety factor was high (Dong et al., 2017). Based on this, we speculated that the above three indirect measurement methods may have more application potential in estimating the VO_2max_ of drug addicts, and we have previously verified the effectiveness of the step test in predicting the VO_2max_ of women methamphetamine addicts (Wang et al., 2020). However, the cardiorespiratory endurance of body will have gender differences to a large extent, and it was currently unknown which method was more effective in estimating the VO_2max_ of men drug addicts.

In addition, from the perspective of drug users, the type of drug use and the years of drug use seem to be the two important study variables (Chen Y. L. et al., 2017; Wang et al., 2019). Therefore, based on the previous study, this study adopted the comparative analysis of 2 (chemically synthesized and natural plant-derived drug addicts) × 3 (30-s leg elevation, 1,000 m walking, and step test) to find out the most suitable indirect measurement method of VO_2max_ for men drug addicts of different types of drug addicts and provide a theoretical and practical reference for the future development of the exercise rehabilitation prescriptions.

## Materials and Methods

### Subjects

A total of 38 men drug addicts were recruited voluntarily in the Xiyanping Education, Correction and Addiction Treatment Center in Chongqing, China. A total of 30 people were finally determined to participate in the trial through the following inclusion and exclusion criteria: (1) between 18 and 50 years old, (2) currently in a state of imprisonment and has undergone compulsory withdrawal rehabilitation for more than 3 months, (3) education level was elementary school and above, (4) diagnosis through structured interviews meets the DSM-IV evaluation criteria for drug-dependent persons, (5) no history of mental illness or any immediate family members suffering from mental illness, (6) no heart or cardiovascular disease, and (7) no physical disability or no aerobic exercise of moderate-intensity or above. In terms of demographic characteristics, the age (*P* < 0.001) and the years of drug use (*P* < 0.01) of natural plant-derived drug addicts were significantly higher than those of chemically synthesized drug addicts, and there was no significant difference in height, weight, BMI, and resting heart rate between the two (*P* > 0.05). See Table 1 for details.

**Table 1:** The demographic characteristics of subjects.

**Items**	**Chemically synthesized** **drug addicts** **(*n* = 15)**	**Natural plant-derived** **drug addicts** **(*n* = 15)**	***P***
Age (years)	28.13 ± 4.93	36.67 ± 4.12	<0.001
Height (m)	1.66 ± 0.99	1.67 ± 0.54	0.829
Weight (kg)	68.31 ± 11.37	64.81 ± 7.95	0.337
BMI	24.68 ± 2.63	23.39 ± 2.56	0.186
Years of drug use (year)	4.27 ± 1.58	6.80 ± 2.21	0.001
Resting heart rate (beats)	78.60 ± 7.60	77.53 ± 6.63	0.685

“*Chemically synthesized drug” mainly refers to methamphetamine, ecstasy, and other amphetamine chemically synthesized drugs, and “natural plant-derived drug” mainly refers to drugs extracted from natural plants such as heroin, morphine, and cannabis (Xue, 2010)*.

### Research Procedure

All the 30 subjects who were screened and included in the trial signed the informed consent form after they were aware of the content and requirements of this study. Then, they completed the five sequence tasks, namely baseline test, 30-s high-leg lift, 1,000-m walk, 3-min step test, and CPX (except for the baseline test and CPX, the other three tests were in random order), with more than 24 h between each task test. It should be pointed out that since the CPX requires the subject to pedal to the exhausted state, the subject should rest for more than 72 h before performing the test. All experimental tasks are scheduled to be conducted from 9:00 a.m. to 11:30 a.m. or 2:00 p.m. to 4:00 p.m. every day.

1. Baseline test. Background information, such as demographic data, drug use history, drug dependence survey, and physical fitness indicators in a quiet state, such as blood pressure, height, and weight.
2. Thirty-second high-leg lift. When raising the legs on the spot, the subjects were required to look straight ahead with both eyes, keep the upper body straight, alternately raise the legs to level, with the thighs and the abdomen at a 90° angle, and swing the arms naturally in a running posture. During the experiment, subjects need to raise their legs *in situ* for the 30 s at the fastest speed. The time was controlled by a recorder with a stopwatch, and another recorder was responsible for recording the number of the subjects who raised their legs in 30 s (i.e., lifting of the left or right leg was considered to be completed once). Finally, the total number of leg lifts was used as a reference index for estimating VO_2max_.
3. 1,000-m walk. The subjects were required to swing their arms vigorously in strides and quickly walk 1,000 m on the outdoor track and field, and they were not allowed to rest or walk slowly in the task. Throughout the test, the tester used a stopwatch to record the time it took for the subject to effectively complete the 1,000 m. Finally, the total time consumed (seconds) was used as a reference index for estimating VO_2max_.
4. 3-min step test. The subjects continued to go up/down a 40-cm step at a fixed speed of 30 times/min (one up and down is one time) within 3 min, and in the process of climbing the steps, a metronome was used to give the auditory feedback for the subjects to control the pedaling rhythm. As soon as the 3-min step-up was completed, the subject immediately sat on the stool to get relaxed and adjusted his/her breathing, and a step tester was used to measure the heart rates of the subject thrice during the recovery period. After the data collection was completed, the step test was ended. It should be pointed out that if the stepping rhythm is slow for three consecutive beats during the step test, the stepping test will be stopped immediately.
5. Cardiopulmonary exercise test. After the subjects put on the test equipment, they sat upright on a stationary power bicycle. The load plan was as follows: the first 2 min were no-load exercise (0 w), then the exercise load is linearly increased by 10 w/min, and the subject needs to maintain the speed of 6070 r/min to pedal the power bicycle. During this period, if the pedaling rhythm was <60 r/min for 3 s, stop pedaling immediately. During the test, the equipment system will synchronously and continuously record the gas metabolism parameters of the subject, such as VO_2_, at each moment, and the subject will continue to be verbally motivated throughout the process until reaching a state of exhaustion [i.e., in line with the stop motion conditions of American College of Sports Medicine (ACSM, 2013)]. During the entire pedaling process, testers will be there to provide safety protection and supervision to prevent falling.

It is noted that in the above five tests, if the subject does not complete or completes any of the tests as required, then all the test data of the subject will not be included in the final statistical analysis. Meanwhile, in this study, based on the recommendations of the ACSM, the test should be stopped when the subject has the following symptoms during the test: (1) the subject voluntarily asks to stop exercising, (2) the subject has symptoms such as dizziness, nausea, pale face, difficulty in breathing, and weakness in limbs, (3) abnormal heart rate, such as too fast or too slow, and (4) VO_2_ reaches the plateau (i.e., a VO_2_ growth rate was <150 ml/min). The heart rate of the subject during exercise was monitored by the SUUNTO Smart Sensor Bluetooth heart rate belt. This study has been approved by the Ethics Review Committee of Southwest University Hospital in China (No. 201905).

### Measuring Tools

#### Power Bike

The stationary power bicycle (Lode-906900) made in the Netherlands was used for pedaling. The subjects could adjust the height of the seat by themselves, and control the speed of the power car by pedaling rhythm. The heart rate belt was connected *via* Bluetooth on the power car screen, which will display the changes in the real-time heart rate of the subject.

#### Portable Cardiopulmonary Function Telemeter

A portable cardiopulmonary function telemeter (PKSP-11, China) made in China was used to monitor the respiratory indicators of subjects, such as VO_2max_, and the subject needs to wear a mask and a gas analyzer. Before each test, the equipment was calibrated with standard gases (i.e., 15% oxygen, 5% carbon dioxide, and 80% nitrogen) and with a special capacity calibration cylinder (Cortex-11, Germany). The changes in the cardiopulmonary function indicators of the subject during exercise were simultaneously monitored, and the gas exchange of the subject during the entire exercise process was collected.

#### Step Tester

The China-made TZCS-3 step tester was used to measure the step index, and the heart rate was tested by infrared detection of the blood flow changes of the finger pulse, which was convenient to use and high in accuracy. The subject sits on the chair in a relaxed posture and puts the middle finger into the finger pulse test clip with the palm down. After a few seconds, the indicator light on the finger pulse clip will start flashing according to the pulse. For a total of three tests, each lasting for 30 s (with an interval of 30 s), the flashing of the signal light was synchronized with the heartbeat of the subject, and the heart rate and step index of the recovery period were automatically generated after 3.5 min.

#### Other

In addition, there was a portable step (40 cm, China), electronic blood pressure meter (RS800CX, Finland), stopwatch (ZSD-013, Xingluda, China), and other small test tools.

### Statistical Analysis

The SPSS 21.0 statistical software was used to process and analyze the data. First, the independent sample *t*-test was used to test the index differences between different types of drug addicts. Second, the Pearson's correlation analysis was used to test the correlation between the variables, and then linear regression analysis was used to test the estimated effect power. Finally, the Bland Altman chart was used to test the level of agreement between the estimated value and the true value. After the Shapiro–Wilk test, all the data were collected using the normal distribution (*P* > 0.05), and the parameter significance level was set to α = 0.05.

## Results

### The Difference Analysis of the Test Results of Different Drug Addicts

Table 2 shows that the different types of drug addicts have significant differences in many test indicators. Among them, the years of drug addicts and the walking time of 1,000 m of natural plant-derived drug addicts were significantly higher than those of chemically synthesized drug addicts (*t* = −3.61, *P* = 0.001 and *t* = −3.00, *P* = 0.006, respectively). The VO_2max_, 30-s high-leg raises, and 3-min step index of chemically synthesized drug addicts were significantly higher than those of natural plant-derived drug addicts (*t* = 3.17, *P* = 0.004; *t* = 2.50, *P* = 0.018; and *t* = 3.14, *P* = 0.004, respectively).

**Table 2:** Statistics of test data of subjects (*n* = 30, mean ± SD).

**Items**	**Chemically** **synthesized** **(*n* = 15)**	**Natural** **plant-derived** **(*n* = 15)**	***t***	***P***
Years of drug use (year)	4.27 ± 1.58	6.80 ± 2.21	−3.61	0.001
VO_2max_ (ml/min/kg)	30.73 ± 2.31	27.40 ± 3.36	3.17	0.004
30 s high leg raises (frequency)	85.13 ± 7.06	79.00 ± 6.35	2.50	0.018
1000 m walking time (min)	9.01 ± 0.57	9.74 ± 0.75	−3.00	0.006
3 min step index	56.37 ± 5.28	48.54 ± 8.07	3.14	0.004

“*VO_2max_” refers to the true maximal oxygen uptake value obtained through the CPX*.

### Correlation Analysis Between VO_2max_ Predictive Indicators and Measured Indicators

Among chemically synthesized drug addicts (Table 3), the VO_2max_ was significantly negatively correlated with the years of drug use and walking time (*r* = −0.66, *P* = 0.007; *r* = −0.64, *P* = 0.009, respectively), while the VO_2max_ was significantly positively correlated with the number of high-leg raises and the step index (*r* = 0.55, *P* = 0.034; *r* = 0.74, *P* = 0.002, respectively). Among natural plant-derived drug addicts (Table 4), the VO_2max_ was significantly negatively correlated with the years of drug use and the walking time (*r* = −0.63, *P* = 0.011; *r* = −0.61, *P* = 0.016, respectively), while the VO_2max_ was significantly positively correlated with the number of high-leg raises and the step index (*r* = 0.64, *P* = 0.009; *r* = 0.77, *P* = 0.001, respectively). The correlation among the main variables was significant, which provides a theoretical basis for the subsequent regression analysis (see Table 3 for details).

**Table 3:** Correlation matrix of chemically synthesized drug addicts (*n* = 30).

	**Years of** **drug use**	**VO**_**2max**_	**High leg** **raises**	**Walking** **time**	**Step** **index**
Years of drug use	1				
VO_2max_	−0.66^**^	1			
High leg raises	−0.46	0.55^*^	1		
Walking time	0.64^**^	−0.64^**^	−0.59^*^	1	
Step index	−0.53^*^	0.74^**^	0.52^*^	−0.43	1
Mean	4.27	30.73	85.13	9.01	56.37
SD	1.58	2.31	7.06	0.57	5.28

* *P < 0.05„*

** *P < 0.01*.

**Table 4:** Correlation matrix of natural plant-derived drug addicts (*n* = 30).

	**Years of** **drug use**	**VO**_**2max**_	**High leg** **raises**	**Walking** **time**	**Step** **index**
Years of drug use	1				
VO_2max_	−0.63^*^	1			
High leg raises	−0.31	0.64^**^	1		
Walking time	0.59^*^	−0.61^*^	−0.59^*^	1	
Step index	−0.52^*^	0.77^**^	0.42	−0.64^**^	1
Mean	6.80	27.40	79.00	9.73	48.54
SD	2.21	3.36	6.35	0.75	8.07

* *P < 0.05,*

** *P < 0.01*.

### Linear Regression Analysis of Different Methods for Estimating VO_2max_ of Drug Addicts

#### Unary Linear Regression Analysis

In this study, the real measurement of the VO_2max_ was used as the dependent variable, and the independent variables were the 30-s high-leg raises, the 1,000-m walking time, and the 3-min step index to establish a linear regression equation.

**Figure 1:**
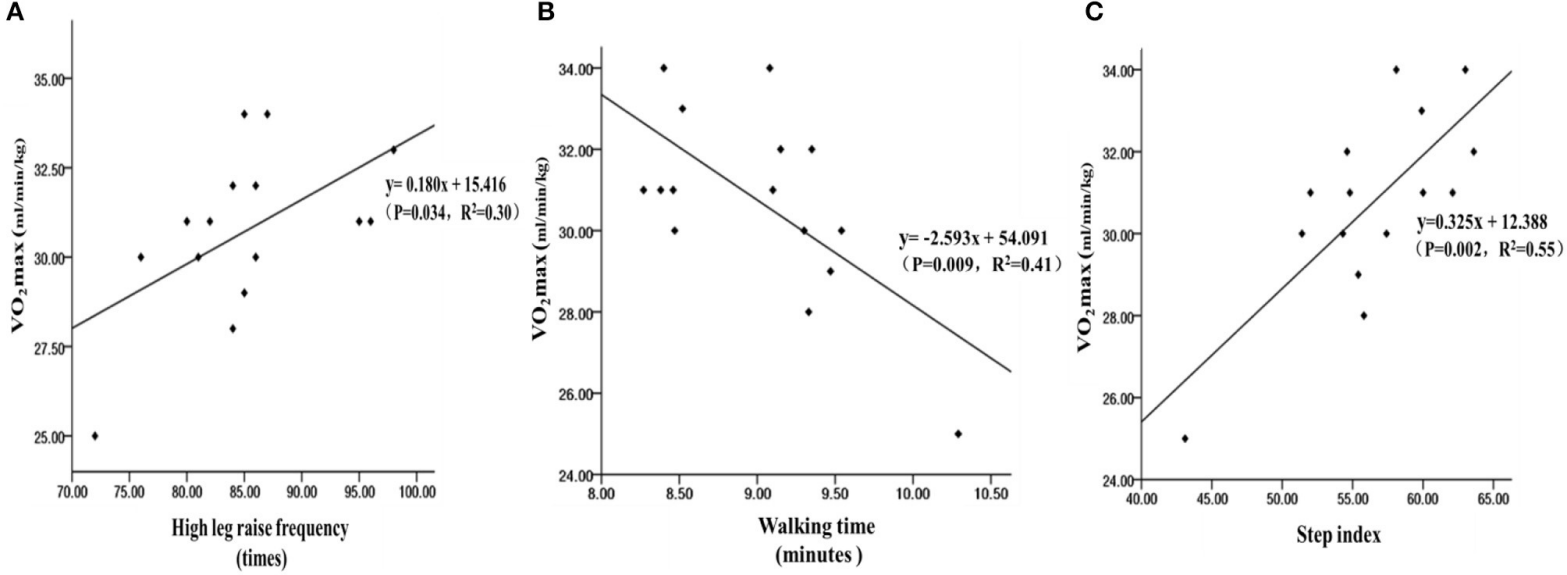
The linear regression scatter plot of the 30-s high-leg raises **(A)**, 1,000-m walking time **(B)**, and 3-min step index **(C)** to estimate the VO_2max_ of chemically synthesized drug addicts.

Among chemically synthesized drug addicts, the 30-s high-leg lift could effectively predict the VO_2max_ (*R* = 0.55, *R*^2^ = 0.30, *P* = 0.034, Figure 1A), the 1,000-m walking could effectively predict the VO_2max_ (*R* = 0.64, *R*^2^ = 0.41, *P* = 0.009, Figure 1B), and the 3-min step test could also effectively predict the VO_2max_ (*R* = 0.74, *R*^2^ = 0.55, *P* = 0.002, Figure 1C). From the results, the three methods could effectively estimate the VO_2max_ of chemically synthesized drug addicts to varying degrees. Among them, the determination coefficient *R*^2^ of 3-min stepping was the highest, indicating that the 3-min step test has the best predictive effect.

**Figure 2:**
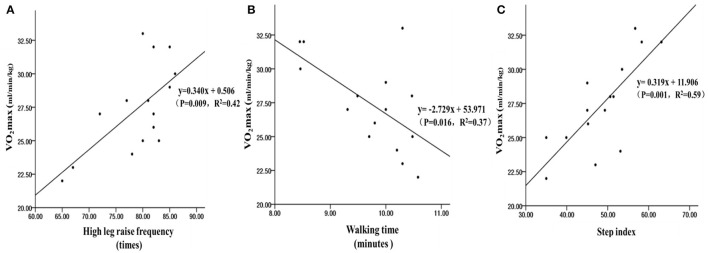
The linear regression scatter plot of the number of 30-s high-leg raises **(A)**, 1,000-m walking time **(B)**, and 3-min step index **(C)** to estimate the VO_2max_ of natural plant-derived drug addicts.

Among natural plant-derived drug addicts, the 30-s high-leg lift could effectively predict the VO_2max_ (*R* = 0.64, *R*^2^ = 0.42, *P* = 0.009, Figure 2A), the 1,000-m walking could effectively predict the VO_2max_ (*R* = 0.61, *R*^2^ = 0.37, *P* = 0.016, Figure 2B), and the 3-min step test could also effectively predict the VO_2max_ (*R* = 0.77, *R*^2^ = 0.59, *P* = 0.001, Figure 2C). From the results, the three methods could effectively estimate the VO_2max_ of natural plant-derived drug addicts to varying degrees. Among them, the determination coefficient *R*^2^ of 3-min stepping was the highest, indicating that the 3-min step test has the best predictive effect.

#### Multiple Linear Regression Analysis

This study combined the years of drug use to explore more in-depth methods of estimating the VO_2max_ of drug addicts (Table 5). Among chemically synthesized drug addicts, the years of drug use could effectively estimate the VO_2max_ (Equation 1), and the regression equation was as follows: *y* = −0.663*x* + 34.878 (*R*^2^ = 0.44, *P* = 0.007). On this basis, we added the number of high-leg lifts as an independent variable and found that the years of drug use combined with the number of high-leg lifts could not effectively estimate the VO_2max_ (Equation 2). Similarly, the years of drug use combined with walking time could not effectively estimate the VO_2max_ (Equation 3). However, this study found that the years of drug use (*x*_1_) combined with the step index (*x*_2_) could effectively estimate the VO_2max_ of men drug addicts (Equation 4), and the regression equation was as follows: *y* = −0.551*x*_1_ + 0.238*x*_2_ + 19.647 (*R*^2^ = 0.65, *P* = 0.019). Among natural plant-derived drug addicts, the years of drug use could effectively estimate the VO_2max_ (Equation 5), and the regression equation was as follows: *y* = −0.962*x* + 33.942 (*R*^2^ = 0.40, *P* = 0.011). On this basis, we added the number of high-leg lifts as an independent variable and found that the years of drug use (*x*_1_) combined with the number of high-leg lifts (*x*_2_) could effectively estimate the VO_2max_ (Equation 6), and the regression equation was as follows: *y* = −0.732*x*_1_ + 0.263*x*_2_ + 11.631 (*R*^2^ = 0.63, *P* = 0.020). The years of drug use combined with walking time could not effectively estimate the VO_2max_ (Equation 7). However, this study found that the years of drug use (*x*_1_) combined with the step index (*x*_3_) could effectively estimate the VO_2max_ of men drug addicts (Equation 8), and the regression equation was as follows: *y* = −0.488*x*_1_ + 0.250*x*_3_ + 18.598 (*R*^2^ = 0.67, *P* = 0.009).

**Table 5:** Stepwise regression of different methods to estimate VO_2max_ (*n* = 30).

**Variable**	**Chemically synthesized drug addicts**				**Natural plant-derived drug addicts**			
	**Equation 1**	**Equation 2**	**Equation 3**	**Equation 4**	**Equation 5**	**Equation 6**	**Equation 7**	**Equation 8**
Constant	34.878	25.334	46.821	19.647	33.942	11.631	47.373	18.598
Years of drug use	−0.663^**^	−0.763^*^	−0.623	−0.551^*^	−0.962^*^	−0.732^*^	−0.641	−0.488^*^
High leg raises		0.102				0.263^*^		
Walking time			−1.491				−1.604	
Step index				0.238^*^				0.250^**^
R	0.66	0.72	0.72	0.81	0.63	0.79	0.70	0.82
R^2^	0.44	0.52	0.52	0.65	0.40	0.63	0.48	0.67
*F*	10.21^**^	6.40^*^	6.50^*^	11.29^**^	8.73^*^	10.02^**^	5.63^*^	11.93^***^

* *P < 0.05,*

** *P < 0.01,*

*** *P < 0.001. The coefficients in the table were nonstandardized β values*.

### Reliability and Validity Analysis of Different Methods for Estimating VO_2max_ of Drug Addicts

To clarify the effectiveness of different methods for estimating VO_2max_, we separately calculated the estimation accuracy of each available method (Table 6). The results showed that: (1) among the chemically synthesized drug addicts, whether it was a unary linear regression or multiple linear regression, the estimation accuracy of the step index was higher. In comparison, the VO_2max_ estimation accuracy of chemically synthesized drug addicts based on the years of drug use combined with the step index was higher (96.48%). (2) Among natural plant-derived drug addicts, the estimation accuracy of the unary linear regression equation established by the step index was the highest (94.30%), the years of drug use combined with the times of high-leg lifts and the years of drug use combined with the step index were second, and the estimation accuracy of both was 94.03%.

**Table 6:** Accuracy statistics of different methods to estimate the VO_2max_ (*n* = 30).

	**Chemically synthesized drug addicts**				**Natural plant-derived drug addicts**			
	**Regression equation**	***R^**2**^***	**95%CI**	**Accuracy (%)**	**Regression equation**	***R^**2**^***	**95%CI**	**Accuracy (%)**
A	y = 0.180x+15.416	0.30	(0.23, 0.34)	94.81	y = 0.340x+0.506	0.42	(0.40, 0.45)	92.89
B	y = −2.593x+54.091	0.41	(0.39, 0.45)	95.16	y = −2.729x+53.971	0.37	(0.34, 0.41)	92.79
C	y = 0.325x+12.388	0.55	(0.52, 0.61)	95.60	y = 0.319x+11.906	0.59	(0.56, 0.62)	94.30
A+D	–	–	–	–	y = −0.732x_1_+0.263x_2_ +11.631	0.63	(0.61, 0.66)	94.03
C+D	y = −0.551x_1_+0.238x_2_ +19.647	0.65	(0.62, 0.68)	96.48	Y = −0.488x_1_+0.250x_2_ +18.598	0.67	(0.65, 0.70)	94.03

*The estimated accuracy = 1 – [(cardiopulmonary exercise test measured value – estimated value)/cardiopulmonary exercise test measured value] ×100%. “A” means “high-leg lifts,” “B” means “walking time,” “C” means “step index,” and “D” means “years of drug use.”*

**Figure 3:**
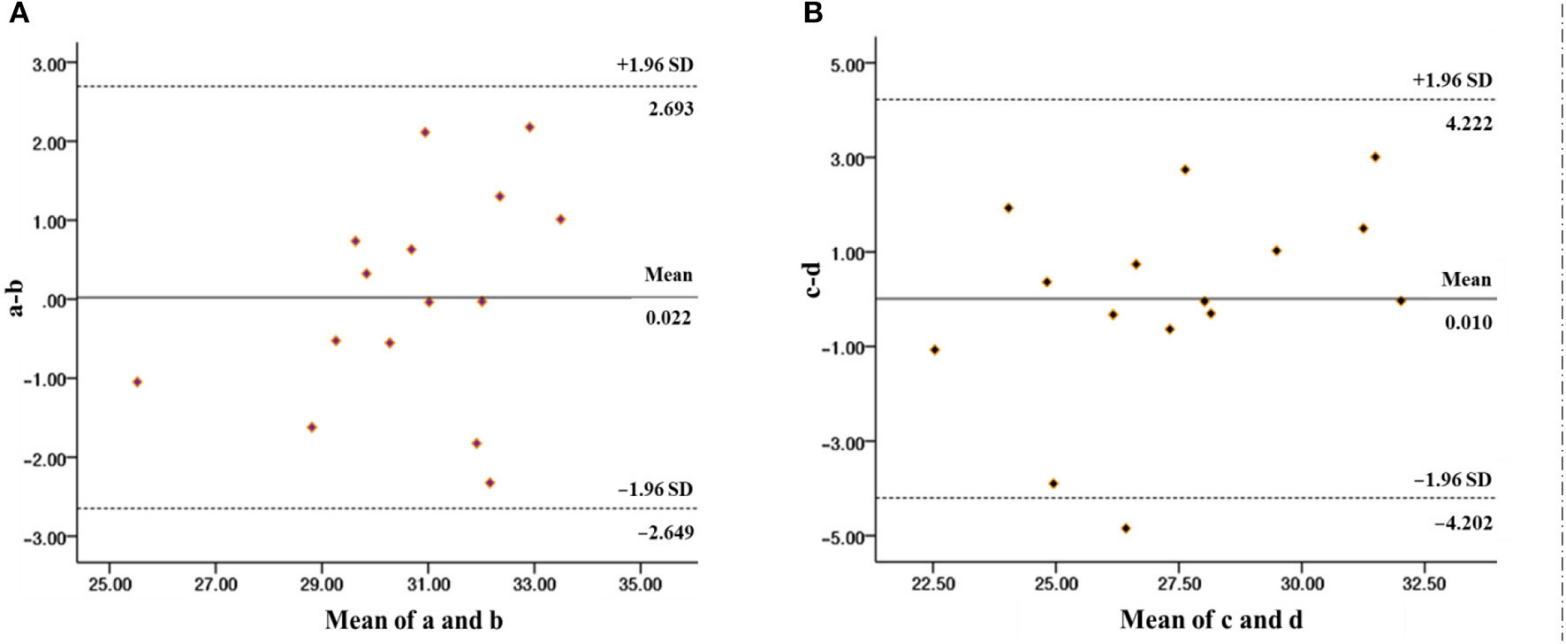
Bland Altman diagrams of different methods for estimating the VO_2max_ of chemically synthesized drug addicts **(A)** and natural plant-derived drug addicts **(B)**. Note: “a” in **(A)** represents the difference between the VO_2max_ measured by C PX and the VO_2max_ estimated by the years of drug use combined with the step index, and “b” means the mean value of the two. “c” in **(B)** represents the difference between the VO_2max_ measured by CPX and the VO_2max_ estimated by the step index, and “d” represents the mean value of the two.

To further verify the best estimation method of VO_2max_ for chemically synthesized and natural plant-derived drug addicts, in this study, the Bland Altman diagram was used to evaluate the reliability and validity of the estimation of the VO_2max_ of chemically synthesized drug addicts estimated by the years of drug use combined with the step index (Figure 3A) and the step index to estimate the VO_2max_ of natural plant-derived drug addicts (Figure 3B), respectively. The results showed that the two estimation methods have a good consistency. It was worth noting that the latter has a slight deviation, and we speculated that this may be related to the sample size. Studies have shown that when the sample size was small, the sampling error will be relatively large, and the larger the sample size, the wider the data coverage, the smaller the limits of agreement CI range, the easier it is to get a better consistent conclusion.

## Discussion

This study was the first to systematically explore the method of estimating the VO_2max_ of men drug addicts. We have fully considered the differences in drug use types and estimation methods, and through comparative analysis of different indirect measurement methods, we have, respectively, revealed the best estimation formulas for VO_2max_ of chemically synthesized and natural plant-derived drug addicts. This may provide convenience for the targeted monitoring of the cardiopulmonary function of men addicts of different drug types and then provide a basis and reference for formulating scientific and targeted exercise rehabilitation prescriptions.

We found that there were significant differences in the VO_2max_ of addicts of different types of drugs, and the VO_2max_ of chemically synthesized drug addicts was significantly higher than that of natural plant-derived drug addicts. Previous studies have shown that synthetic chemical drugs such as amphetamine, which mainly act on the central nervous system of the human body, were more likely to cause individual mental damage and cognitive impairment and have strong neurological dependence (Panenka et al., 2013; Wang and Zhu, 2017). Natural plant-derived drug addicts such as marijuana and heroin were mainly involved in damaging the immune function of the body and were more likely to cause damage to the heart, liver, kidney, and other organs of the drug addicts (Hu, 1992; Ye, 1995; Zhu, 2000), and the long-term use of cocaine could lead to decreased lung function (Yang, 2006). It shows that different types of drugs have different degrees of damage to the body of drug users, which shows that the grouping of drug users according to the types of drug use has certain scientific rationality. Meanwhile, compared with synthetic chemical drugs, the natural plant-derived drug addicts were often older, and the latter has a higher number of years of drug use. It can be seen that the age and years of drug use may be two moderating variables for drug damage to the body, and it was manifested as the older and the longer the drug use, the lower the value of VO_2max_. However, previous studies have mainly focused on the difference in the years of drug use, for example, the degree of damage to the body caused by drug use will increase with the increase in the years of drug use (Jiang, 2006; Chen Y. L. et al., 2017). Therefore, the relationship between age and the cardiopulmonary function of drug addicts needs to be further studied. In addition, it should be noted that VO_2max_ plays an important role in assessing the cardiopulmonary function of drug addicts. The VO_2max_ was not only a risk indicator for estimating cardiovascular degradation and monitoring the functional status of the patient (Aspenes et al., 2011) but also an important indicator for assessing the aerobic endurance of the body (Bosquet et al., 2002), and cardiorespiratory endurance such as VO_2max_ was considered to have significant gender differences (Li et al., 2007; Lin et al., 2018). The lower the level of cardiopulmonary function, the higher the risk of premature death, which maintaining a good cardiorespiratory fitness level can reduce the risk and mortality of many chronic diseases (Larsen et al., 2014). In the field of exercise detoxification, VO_2max_ was considered to be one of the important indicators to assess the physical fitness status of drug addicts and the effect of exercise intervention, and the VO_2max_ of drug addicts could be effectively improved through active exercise (Dolezal et al., 2013; Wang and Zhu, 2017), thereby reducing the risk of diseases such as heart disease and hypertension (Henry et al., 2012). Therefore, strengthening the monitoring of VO_2max_ of drug addicts (especially natural plant-derived drug addicts) was particularly important for ensuring their quality of life and formulating targeted rehabilitation training prescriptions.

The results of this study showed that the use of 30-s high-leg lift, 1,000-m walking, and 3-min stepping were all significantly correlated with the VO_2max_ of drug addicts, and the three methods could effectively estimate the VO_2max_ of drug addicts, which indicates that the indirect measurement method was equally effective for drug addicts, and could reflect the level of cardiorespiratory endurance of drug addicts to a certain extent, which was more consistent with previous studies (Li et al., 2010; Dong et al., 2017). Interestingly, we found through comparison that there were certain differences in the best estimation methods of VO_2max_ among different drug addicts. It was shown that multiple linear regression constructed with the years of drug use combined with the step index was more effective and accurate in estimating the VO_2max_ of chemically synthesized drug addicts, while the unary linear regression constructed with step index could more effectively and accurately estimate the VO_2max_ of natural plant-derived drug addicts. Previous studies have shown that the step test has good measurement reliability and could effectively assess the cardiovascular system functions of the body, and the step test index was more sensitive to exercise effects than VO_2max_ (Wang and Deng, 2003). Studies have shown that compared with riding power bikes, up and down steps can mobilize more muscles throughout the body, promote blood oxygen circulation, and increase oxygen consumption (Fan et al., 2019). However, simply relying on the step index to determine VO_2max_ was gradually being questioned. During the test, it was necessary to return to the physiology itself to better estimate VO_2max_ and other aerobic fitness (Ryhming, 1953). This may be because the exercise load conditions of the step test are constantly changing, and the step height, exercise rhythm and duration, and even the calculation method of the step index vary with different test subjects (Buckley et al., 2004). Based on this, many scholars have added the physiological and morphological characteristics of the subject in the process of predicting VO_2max_, hoping to improve the accuracy of predicting VO_2max_ (Wang et al., 2008). From the results, after we combined the years of drug use with the step index, the prediction accuracy of the VO_2max_ for chemically synthesized drug addicts was significantly improved, indicating that the appropriate combination of individual characteristics was, in fact, more conducive to improving the estimation accuracy. Therefore, the important variable of the years of drug use should be fully considered in this study (Jiang, 2006; Chen Y. L. et al., 2017). In natural plant-derived drug addicts, combining the years of drug use and the step index, the estimation accuracy was also better than the estimation accuracy of simple high-leg lift and walking time. However, in comparison, using a single step index to estimate the VO_2max_ of natural plant-derived drug addicts has the highest accuracy. We speculated that this may be related to the influence of different drug types on the physiological characteristics of the addicts and the difference in the years of drug use.

However, since this study was the first to explore the method of estimating VO_2max_ of men drug addicts, there were some limitations. For example, the sample size of this study was relatively small, and the samples were all in Chongqing, China, which easily leads to the lack of universality of the study results. Meanwhile, based on the results of this study, we do not know whether the three estimation methods in this study were equally applicable to female drug addicts. In terms of estimation methods and techniques, in addition to the three methods used in this study, other more scientific and effective methods require follow-up study to discuss them in depth. In addition, future studies can also use more advanced science and technology (such as artificial intelligence, etc.) to improve estimation power.

## Conclusion

Indirect measurement methods such as the 30-s raising legs, 1,000-m walking, and 3-min step experiment could effectively estimate the VO_2max_ of men drug addicts, but there were certain differences between the best estimation methods for chemically synthesized and natural plant-derived drug addicts. From the perspective of prediction accuracy, multiple linear regression constructed by the years of drug use combined with the step index has the highest estimated accuracy for the VO_2max_ of chemically synthesized drug addicts, while the unary regression equation established by a single step index has the highest estimated accuracy for the VO_2max_ of natural plant-derived addicts. Therefore, future studies can fully consider the physical characteristics of drug users and combine more cutting-edge science and technology to make the estimation accuracy of VO_2max_ closer to the real level.

## Data Availability Statement

The original contributions presented in the study are included in the article/supplementary material, further inquiries can be directed to the corresponding author/s.

## Ethics Statement

The studies involving human participants were reviewed and approved by Ethics Committee of Southwest University Hospital. The patients/participants provided their written informed consent to participate in this study.

## Author Contributions

All authors designed this study, contributed and approved the final manuscript. KW and HJ carried out the experiment procedure. XC recruited the individuals with drug addicts. LY and TZ undertook the statistical analysis and graphical representation of the data. JL revised the draft.

## Funding

This study was supported by the Fundamental Research Funds for the Central Universities of China (Grant No. SWU2009103).

## Conflict of Interest

The authors declare that the research was conducted in the absence of any commercial or financial relationships that could be construed as a potential conflict of interest.

## Publisher's Note

All claims expressed in this article are solely those of the authors and do not necessarily represent those of their affiliated organizations, or those of the publisher, the editors and the reviewers. Any product that may be evaluated in this article, or claim that may be made by its manufacturer, is not guaranteed or endorsed by the publisher.
